# The British Medical Association Meeting

**Published:** 1914-08-01

**Authors:** 


### The British Medical Association Meeting.

The Aberdeen meeting of the British Medical Association began on July 25, and the medico-political topics discussed are of greater importance than anything the Association has had to decide since the famous Insurance Act debates. So highly controversial were the subjects dealt with that they were debated in private, and condensed reports are all that at the moment of writing are available for examination.

To begin with, it was proposed to establish a fund to compensate such practitioners as may suffer pecuniary loss through loyalty to the policy of the Association. In principle this scheme is sensible enough; though after the disloyalty and cowardice exhibited at the time of stress in December 1912 and January 1913, it seems hardly likely that such loyalty is to be expected. In other words, the members betrayed themselves, each other, and the Association a year and a half ago: so that there is none of the mutual confidence and trust now existing amongst them, such as is necessary before sacrifices for the common interest will be contemplated in the future. Nor must it be overlooked that large numbers of medical men outside the profession contributed to the guarantee fund raised two years ago, but are little likely to do so again after what happened then. However, to let that pass for the present, the policy of raising a defence fund was, after some criticism and opposition, adopted by the meeting.

Then came a more crucial matter—namely, how the fund is to be protected. The Brighton Division brought up a motion directing that the administration of it should be in the hands of trustees. To this plan the alternative policy was preferred by many speakers of registering as a trade union. The advocates of this method urged that the fund is practically for strike or lock-out pay. It is being formed chiefly in view of future developments in National Insurance administration or legislation which may conceivably be injurious to the interests of panel doctors. If, for instance, within the next few years new regulations and conditions of employment under the Insurance Act should be imposed on medical men, prejudicial to the interests of the profession, this fund would undoubtedly afford the means of making opposition to the new terms effective by guaranteeing support to those members who may be dependent upon panel practice for their livelihood. It would also be available if the interests of any particular class —as for example, school doctors, Poor-Law medical officers, public health officials, institutional medical staffs, and so forth—were attacked or jeopardised.

The arguments in favour of the trade-union solution of the problem were effectively presented by Dr. Buttar, chairman of the Special Fund Committee. He pointed out that the fund was bound to be used for objects which would be held to be " in restraint of trade " and should therefore be protected against actions for damages for tort. Such protection is secured to trade unions by the Trades Disputes Act, but is not secured by the establishment of a trust. The opposite view was upheld by Dr. Beaton, who was supported by a majority of the voters, although the majority report of the Council recommended the trade-union method. It was decided that the fund should be placed in the hands of a Trust Association or other form of organisation, not being a registered trade union. The general view seemed to be that there is something derogatory to the dignity of a profession by going through the formality of registering as a trade union. A much more cogent objection to the formation of a registered trade union can, however, be urged, and was in fact put forward at the meeting—namely, that medical men are so scattered, and so often in antagonism to the private interests of their colleagues, that unity of action such as a trade union is able to secure by methods of terrorism is out of the question.

### Monday's Sitting.

The second sittings of the Association on July 27 were devoted to matters of almost equal importance to those discussed on July 25; practically the whole debate dealt with questions more or less closely related to National Insurance and its effects upon the future of medical practice in this country. The question of the receipt by visiting staff doctors at institutions of a share in moneys paid to such institutions by the State for the care and treatment of insured persons was thrashed out at some length. On the one hand, it can be shown that the Association and the medical profession has definitely adopted the principle that medical men should not give gratuitous service to those for whom the State assumes responsibility. On the other hand, it was argued, reasonably enough, that to accept such payments means ultimately Government control of the voluntary hospitals, or rather the break-up of the voluntary system as it is now known. The actual discussion raged round a paragraph in the report of the National Insurance Act Committee referring to voluntary tuberculosis dispensaries and institutions; but the principle at stake obviously enough affects all kinds of voluntary hospitals. A Birmingham speaker said that in all the large industrial centres the doom of the voluntary system has been already sounded. Whether or not this assertion influenced the meeting, at all events the National Insurance Act Committee found itself supported by the meeting, and the principle of participation by visiting medical staffs in public money was duly approved.

Compared with these vital matters, the rest of the sitting was of minor interest, and was mainly devoted to attempts to reconcile the irreconcilable—namely, to further the interests of panel practitioners without injuring those of non-panelites, and *vice versa*. The chief reflection left after a study of the accounts of the proceedings is depressing; for the conviction is deepened that the Representative Body of the Association is unfit by temperament or constitution to discuss large issues with wisdom and foresight.

